# Low-Grade Inflammation, Oxidative Stress and Risk of Invasive Post-Menopausal Breast Cancer - A Nested Case-Control Study from the Malmö Diet and Cancer Cohort

**DOI:** 10.1371/journal.pone.0158959

**Published:** 2016-07-08

**Authors:** Joana A. Dias, Gunilla N. Fredrikson, Ulrika Ericson, Bo Gullberg, Bo Hedblad, Gunnar Engström, Signe Borgquist, Jan Nilsson, Elisabet Wirfält

**Affiliations:** 1 Nutritional Epidemiology research group, Department of Clinical Sciences, Lund University, Malmö, Sweden; 2 Experimental Cardiovascular Research, Department of Clinical Sciences, Lund University, Malmö, Sweden; 3 Diabetes and Cardiovascular Disease – genetic epidemiology, Department of Clinical Sciences, Lund University Diabetes Center, Lund University, Malmö, Sweden; 4 Cardiovascular Epidemiology research group, Department of Clinical Sciences, Lund University, Malmö, Sweden; 5 Division of Oncology and Pathology, Department of Clinical Sciences, Lund University, Lund, Sweden; Hunter College, UNITED STATES

## Abstract

**Objective:**

Although cancer promotes inflammation, the role of inflammation in tumor-genesis is less well established. The aim was to examine if low-grade inflammation is related to post-menopausal breast cancer risk, and if obesity modifies this association.

**Methods:**

In the Malmö Diet and Cancer cohort, a nested case-control study was defined among 8,513 women free of cancer and aged 55–73 years at baseline (1991–96); 459 were diagnosed with invasive breast cancer during follow-up (until December 31^st^, 2010). In laboratory analyses of blood from 446 cases, and 885 controls (matched on age and date of blood sampling) we examined systemic inflammation markers: oxidized (ox)-LDL, interleukin (IL)-1β, IL-6, IL-8, tumor necrosis factor (TNF)-α, white blood cells, lymphocytes and neutrophils. Odds ratios (OR) and 95% confidence intervals (CI) for breast cancer risk was calculated using multivariable conditional logistic regression.

**Results:**

Inverse associations with breast cancer were seen in fully-adjusted models, for 2^nd^ and 3^rd^ tertiles of ox-LDL, OR (95% CI): 0.65 (0.47–0.90), 0.63 (0.45–0.89) respectively, *p*-trend = 0.01; and for the 3^rd^ tertile of TNF-α, 0.65 (0.43–0.99), *p*-trend = 0.04. In contrast, those in the highest IL-1β category had higher risk, 1.71 (1.05–2.79), *p*-trend = 0.01. Obesity did not modify associations between inflammation biomarkers and breast cancer.

**Conclusion:**

Our study does not suggest that low-grade inflammation increase the risk of post-menopausal breast cancer.

## Introduction

According to the World Cancer Research Fund International, breast cancer is the commonest cancer in women worldwide, accounting for a total of 1.7 million new cases diagnosed in 2012 [1]. Lifelong over-exposure to sex hormones such as estrogen is believed to be a major factor responsible for the development of post-menopausal breast cancer [2–8].

It is accepted that low-grade inflammation is part of the pathogenesis of several chronic diseases such as cardiovascular diseases (CVD), type 2 diabetes mellitus, and of certain types of cancer [9–11]. Although it is estimated that up to 20% of all cancers arise in association with chronic inflammation, its role in tumor-genesis may not be as clear as it is in CVD [11, 12]. Inflammation is proposed to influence several phases of cancer development, including both initiation and progression [13]. Specifically, chronic inflammation is responsible for promoting the increase of free radicals (ROS) stimulating oxidative stress. During that process several pro-inflammatory cytokines are released, which could influence DNA methylation and create an inflammatory micro-environment with an important role in several steps needed to transform a normal cell into a carcinogenic cell [14]. In the progression, the carcinogenic cells will also feedback positively into the loop of increased low-grade inflammation [15].

Obesity (i.e., body and abdominal fatness) is known to increase the risk of several cancers [2]. Obesity associated inflammation (i.e., adipose inflammation [16]) may partially explain the association between obesity and risk of post-menopausal breast cancer through two possible mechanisms: inflammatory pathways and dysregulated metabolism [17, 18].

Examples of inflammatory mediators are pro-inflammatory cytokines (such as tumor necrosis factor (TNF)-α, interleukin (IL)-1β, IL-6), chemokines, leptin and adiponectin. These potentially have important roles in the development of obesity associated breast cancer [19, 20]. Breast cancer cells respond to higher circulating levels of these pro-inflammatory cytokines by increasing the expression of P450 aromatase [21]. Additionally, ox-LDL is a marker for oxidative stress, and an important cause of vascular inflammation in cardiovascular disease [22].

Although there is a great deal of evidence describing the influence of low-grade inflammation and specifically of cytokines in the promotion, angiogenesis, and metastasis of breast cancer [23–25] their importance in the onset of breast cancer has not previously been extensively examined in humans [26]. Thus, the aims of this study are to examine how several markers of low-grade inflammation and oxidative stress are associated with the risk of post-menopausal breast cancer in women from the Malmö Diet and Cancer (MDC) cohort, and to study if obesity modifies these associations.

## Materials and Methods

### Study design

The Malmö Diet and Cancer study (MDC) is a prospective population-based study. The baseline examinations took place between 1991 and 1996 in Malmö, Sweden. The source population invited to participate was all men and women born between 1923 and 1945 and women until 1950 (74,138 persons). The cohort and the recruitment procedures details have been published elsewhere [27]. The baseline examinations consisted of an extensive questionnaire regarding socio-economic and lifestyle factors, blood sampling and anthropometric measurements performed by trained nurses, and a modified diet-history method, combining a 7-day menu book (for registration of cooked lunch/dinner meals, alcohol, medication and nutrient supplements), a 168-item questionnaire (of other foods), and a 1-h diet history interview. In total, 17,035 women (of a total of 28,098 participants) completed all baseline examinations. The Ethical Committee at Lund University approved the MDC study (LU 51–90). At their first visit to the study center all participants were given detailed information about the Malmö Diet and Cancer study, and gave their written informed consent.

### Study population

This nested case-control study includes women aged 55–73 years at baseline. The age criteria of inclusion (≥ 55 years) intended to minimize the probability of selecting pre-menopausal women. Women with other prevalent cancers, except cervix cancer *in situ*, were excluded. As a result a total of 8,513 women were considered for the study. Of these, 459 women were diagnosed with invasive breast cancer during follow-up (until 31^st^ of December 2010).

Three controls per case (n = 1,377) were randomly selected (from the same source of 8,513 women) and matched on age at baseline (±3 months) and date of blood sampling (±1 month). As additional requirements: controls had to be alive at the time of matching, living in Sweden and free of breast cancer. Plasma samples were available and laboratory analysis of the inflammation markers was successful in 446 cases, and in 910 controls. However, the analytical sample consisted of 446 cases and 885 controls, due to the conditional logistic regression procedure, which included only controls matched with the specific cases. Seven of the cases had only one control with successful laboratory analysis, and the remaining eighteen controls had been matched with cases excluded previous to statistical analysis due to unsuccessful laboratory analysis.

### Case ascertainment

Data on case definition was provided by the Swedish Cancer Registry and the Southern Swedish Regional Tumor Registry until the end of follow-up, 31^st^ of December 2010. Women who were diagnosed with invasive breast cancer during follow-up were considered cases, and invasive breast cancer was defined as all cancers except *in situ* cancer. Information on vital status was obtained from the Swedish Cause of Death Register (of the National Board of Health and Welfare), and from the Total Population Register (of Statistics Sweden).

### Biomarkers of inflammation

Blood samples were drawn from the participants during their first visit to the study center (at baseline). The leukocyte counts were assessed at this point in time using fresh heparinized blood, and a SYSMEX K100 automatic counter (Sysmex Europe, Norderstedt, Germany) [28]. Leukocyte counts were expressed as total (white blood cells, WBC) and differential (i.e. neutrophils, and lymphocytes). Blood components were separated; plasma and serum were frozen at -80°C and stored to be analyzed at a later date.

The biochemical analyses of the other inflammation markers were conducted during the autumn of 2011. Plasma samples were thawed and the concentration of ox-LDL in plasma was analyzed with an ELISA (Mercodia, Uppsala, Sweden), which is a capture ELISA using the mAb-4E6 antibody against a conformational epitope in oxidized ApoB-100, developed by Holvoet et al [29]. The inter-assay variation was 5.6% for the ox-LDL ELISA and all samples were within the range of detection. The cytokines, IL-1β, IL-6, IL-8, and TNF-α were analyzed with the Human Pro-inflammatory 4-plex II Ultra-sensitive kit (Meso Scale Discovery, Gaithersburg, MD, USA) [30]. The inter-assay variation was 19%, 17%, 10%, and 19%, respectively. The concentration of IL-1β was below the lower limit of detection (LLOD; varied from 0.00 to 0.57 pg/mL across 18 plates) in about 74% of the samples. The IL-6 concentration was below LLOD (between 0.16 and 0.73 pg/mL) in 1.9% of the samples. In three samples the concentrations of IL-8 and TNF-α were not detectable (LLOD between 0.04 and 0.15 pg/mL and 0.15 and 0.58 pg/mL, respectively). There were no missing values for ox-LDL or the cytokines. All samples were analyzed randomly across the 18 plates. In 73% of the samples (n = 979) cases and matched controls analyzed in the same plate, whereas in 26% of the samples, cases were analyzed in the different plates than the controls.

### Anthropometric, socio-economic and lifestyle variables

Information on birthdate and gender were retrieved from the personal identification number, and age was calculated. The week of blood sampling refers to the screening date in the MDC study, and it ranges from week 0 (on the 22^nd^ of March 1991) to week 287 (on the 24^th^ of September 1996). Based on the dates of measurement, the season (spring, summer, fall, and winter) was noted. Trained nurses measured participants’ height (m), weight (kg) and hip and waist circumferences (cm), at the baseline examinations. Body Mass Index (BMI) was calculated dividing weight by the squared height (kg/m^2^) and categorized into under/normal weight, overweight and obese (BMI < 25, 25–29, and ≥ 30 respectively). Waist-to-hip ratio (WHR) was calculated by dividing the waist by the hip circumferences. It was further divided into tertiles and sextiles.

Also during baseline examinations, participants completed an extensive questionnaire on socio-economic and lifestyle factors. Participants reported the highest education level achieved, which was divided into 4 categories; primary, elementary, high school or university degree. Smoking status was defined as never, former and current smokers (including irregular smokers). A four category variable described the consumption of alcohol. Zero consumers were participants who reported no alcohol consumption both in the general questionnaire and during the diet history assessment (i.e., no consumption in the 7-day food record); low consumers (<15 grams/day), medium (15–30 g/d) and high consumers (>30 g/d) were defined according to cut-offs based on a biological risk assumption [31].

The questionnaire also assessed leisure time physical activity (PA) with a list of 18 different activities, which were adapted from Minnesota Leisure Time Physical Activity [32]. An activity-specific factor was multiplied by the minutes individuals reported having spent on each activity and further summed into a score. The score was then divided into tertiles, from the lowest to the highest level of PA. The ability of the PA questionnaire to predict health-related risks has been previously examined [33].

### Hormonal exposure

Participants reported their age at menarche, age at menopause and age at the birth of the first child in the questionnaire. In addition, they also reported the number of months each child had been breastfed. The time span between age at menarche and age at menopause was computed. We then subtracted the interruptions caused by periods of pregnancies and lactation, and calculated the number of years with menstrual cycles.

Parity was divided into 5 categories; nulliparous (no children), 1, 2, 3, and 4 or more children. Participants reported use (or not) of oral contraceptives (OC), as well as current use of menopausal hormone therapy (MHT). The information on MHT use was retrieved from a questionnaire item regarding medications used on a regular basis, and from the 7-day food record [34].

### Statistical analysis

The baseline characteristics of established risk factors were examined among cases and controls and differences were explored with odds ratios (OR) and 95% confidence intervals (CI) by using conditional logistic regression, in basic models (unadjusted). All significant factors were further introduced in the same model. When studying the baseline characteristics of the controls across tertiles of several inflammation markers, we used the chi-square test to calculate proportional differences in categorical variables, and the ANOVA to examine mean differences (for continuous variables).

In order to address the skewedness of most biomarkers, levels were ranked into tertiles, or transformed logarithmically when necessary. Since the IL-1β variable included many zeros and values below the LLOD, this variable was classified into 4 categories containing: 0 values (n = 340); all values below LLOD except zeros (n = 640); and the values above LLOD (n = 351) were split into two categories above and below the median (0.40 pg/mL) of the distribution (n = 176 and n = 175). In order to enable ln-transformation of the IL-1β variable (for analysis of continuous variables), a small value was added to this variable.

We have examined the correlations between levels of inflammation markers (ln-transformed) and obesity/adiposity measures (BMI and WHR), adjusting for matching factors (age and week of blood sampling), among controls.

We computed OR and 95% CI for breast cancer risk in tertiles of several biomarkers of inflammation such as ox-LDL, IL-1β, IL-6, IL-8, TNF-α, WBC, lymphocytes and neutrophils, using conditional logistic regression, and controlling for matching variables (age and week of blood sampling); model I. A second model was created adding BMI; model II. A third model included additional adjustments for established risk factors for breast cancer which were significantly associated with breast cancer in our project: hormonal factors (MHT, parity) and abdominal obesity (WHR); model III. In addition, a final model also included possible lifestyle confounders such as smoking status, alcohol, physical activity, and education; model IV. The choice of confounders used in the models was guided by the literature, and by its significance in this study sample. We included all biomarkers significantly associated with breast cancer in the same model in order to assess independent risks. Conditional logistic regression models were successful in 446 cases and 885 corresponding controls.

In sensitivity analysis we sequentially excluded women who were diagnosed during the first, second, and third years of follow-up. In an additional step, women with history of CVD (n = 27) and diabetes (n = 52) at baseline were excluded. We also examined if the season at the time of blood sample collection influenced the observed associations: the differences in the controls; the association between season and breast cancer risk; and evaluating if season influenced associations with inflammation markers. Finally, in an additional step all models were also adjusted for batch effect by adding plate number as a covariate in all models.

We examined the potential interactions between the biomarker concentrations and measures of obesity/adiposity (tertiles of BMI and tertiles of WHR, as continuous variables) by including interaction terms in the basic and fully-adjusted models predicting breast cancer risk, using unconditional logistic regression.

The fully adjusted models included 1142 people in the analysis; 146 (11%) of the subjects had missing data in one or more of the covariates, and 68 cases were censored before earliest event in a stratum. In the MHT variable 8.3% (n = 110) had missing information, whereas in the parity 2.5% (n = 33) had missing information. Sixteen individuals had missing information for the PA variable, and all other variables were either complete or had missing only 1 to 4 people. In the MHT and parity variables, the proportion of case/controls was 1/2 and the missing cases were evenly distributed across categories of the other confounders. Thus, we performed complete case analysis. Additionally, we performed an *ad hoc* analysis including women with missing values for the MHT and parity variables in a separate category, including a total of 1300 women in the fully adjusted model

All statistical analyses were performed with IBM SPSS Statistics (version 22.0), and all statistical tests were two-sided and significance level was set at *p*<0.05.

## Results

### Characteristics and risk factors

Cases had higher WHR, tended to be more overweight or obese (Table 1) and had higher alcohol consumption than controls. Similarly, women holding a university degree were at significantly higher risk for post-menopausal breast cancer when compared to women with primary school. Oral contraceptive (OC) and current MHT use was more common among the cases. In contrast, inverse associations were observed for parity; women with more children had lower breast cancer risk. When all significant factors were introduced in the same model, the associations between WHR, BMI and education, and breast cancer were no longer significant.

**Table 1:** Baseline characteristics among breast cancer cases and matched controls 55–73 years of age from the Malmö Diet and Cancer cohort.

	Cases	Controls		
	(n = 446)	(n = 885)		
	Mean ± SD		OR^¥^ (95%CI)	*p*-value^¥^
Age (y)	62.0 ± 4.8	62.0 ± 4.8		(m.v.)
Week of blood sampling	139 ± 77	139 ± 77		(m.v.)
Height (m)	1.63 ± 0.05	1.63 ± 0.06		0.20
Waist-to-hip ratio (WHR)*^§^	0.80 ± 0.07	0.79 ± 0.06		**0.04**
Age at menarche (*a*)^§^	13.6 ± 1.1	13.6 ± 1.1		0.98
Age at menopause (*b*)	50.2 ± 4.8	50.0 ± 4.6		0.27
Age at birth of first child^§^	24.7 ± 1.2	24.4 ± 1.2		0.16
Breastfeeding time (months)^§^	8.4 ± 2.2	8.1 ± 2.3		0.38
Time span between *a* and *b* (y)	36.5 ± 5.0	36.2 ± 4.8		0.35
Menstrual cycles (y)	34.4 ± 5.0	34.0 ± 5.0		0.16
	N (%)			
**Education**				
Primary school	196 (44.2)	442 (50.1)	1 (ref.)	
Elementary school	155 (35.0)	278 (31.4)	1.26 (0.97–1.64)	
High school	18 (4.1)	47 (5.3)	0.87 (0.49–1.55)	
University	74 (16.7)	117 (13.2)	**1.46 (1.03–2.08)**	0.06
**Smoking status**				
Never smoker	215 (48.2)	458 (51.8)	1 (ref.)	
Former smoker	139 (31.2)	240 (27.1)	1.26 (0.96–1.65)	
Current smoker	92 (20.6)	186 (21.1)	1.05 (0.78–1.41)	0.50
**Alcohol consumption**				
Zero consumers	30 (6.7)	77 (8.7)	1 (ref.)	
Low (<15 g/d)	351 (78.7)	686 (77.5)	1.31 (0.83–2.07)	
Medium (15–30 g/d)	49 (11.0)	114 (12.9)	1.15 (0.66–2.01)	
High (>30 g/d)	16 (3.6)	8 (0.9)	**4.92 (1.91–12.7)**	0.06
**Leisure time PA**				
Tertile 1	158 (35.6)	279 (32.0)	1 (ref.)	
Tertile 2	146 (32.9)	292 (33.5)	0.88 (0.67–1.17)	
Tertile 3	140 (31.5)	300 (34.5)	0.82 (0.62–1.09)	0.18
**BMI**				
Under/Normal weight (<25)	178 (39.9)	406 (45.9)	1 (ref.)	
Overweight (25–30)	195 (43.7)	342 (38.7)	**1.29 (1.01–1.67)**	
Obese (>30)	73 (16.4)	136 (15.4)	1.24 (0.89–1.75)	0.09
**Parity (number of children)**				
0	59 (13.6)	99 (11.5)	1 (ref.)	
1	91 (21.0)	185 (21.4)	0.82 (0.55–1.25)	
2	181 (41.7)	351 (40.6)	0.87 (0.60–1.27)	
3	76 (17.5)	142 (16.4)	0.91 (0.59–1.40)	
≥ 4	27 (6.2)	87 (10.1)	**0.52 (0.30–0.89)**	0.11
**Oral Contraceptive**				
No use	262 (58.9)	562 (63.6)	1 (ref.)	
Reported use	183 (41.1)	322 (36.4)	1.24 (0.97–1.58)	0.08
**MHT**				
No use	284 (70.0)	654 (80.2)	1 (ref.)	
Current Use	122 (30.0)	161 (19.8)	**1.80 (1.35–2.42)**	**<0.001**

^¥^The ORs and 95% CI were calculated with conditional logistic regression analysis, in a basic model (unadjusted), the first category was used as reference.

The *p*-values refer to p-for-linear-trend as categorical variables were introduced linearly. The *p*-values for continuous variables were derived from a GLM model using paired data. M.v., matching variables.

^§^Due to the skewedness of these variables, ln-transformation was used, and means and SD values were back transformed.

*Due to very wide confidence intervals, these variables were introduced as continuous sextiles in the model (this did not affect the p-value greatly).

The distribution of the matching factors and possible confounders across categories of the inflammation markers among controls are described in Table 2 (and in S1 and S2 Tables). Age, week of blood sampling, and WHR vary across categories of most biomarkers; participants tended to be older in the highest biomarker tertiles, except for IL-1β, IL-8 and TNF-α where the pattern is the opposite. Participants in the highest tertile of IL-6 concentration were less likely to have a university degree; less likely to be MHT users, and more likely have an alcohol intake below 15 g/day. These participants were also more likely to be current smokers, and to have higher BMI, when compared to the lowest tertile. Participants in the highest tertile of WBC, neutrophils and lymphocytes were more likely to be current smokers. In addition, participants in the highest tertile of ox-LDL, IL-8 and TNF-α were more likely to be overweight or obese. Lastly, participants in the highest tertiles of IL-1β, IL-8, and TNF-α were less likely to be MHT users.

**Table 2:** Characteristics of breast cancer controls (n = 885) across levels of several inflammation markers (ox-LDL, TNF-α, and IL-1β) in the Malmö Diet and Cancer cohort.

		Ox-LDL				TNF-α				IL-1β			
	Tertile 1	Tertile 2	Tertile 3		Tertile 1	Tertile 2	Tertile 3		0	<LLOD	Cat 1	Cat 2	
	(n = 277)	(n = 302)	(n = 306)		(n = 285)	(n = 283)	(n = 317)		(n = 235)	(n = 432)	(n = 111)	(n = 107)	
	Mean ± SD			*p*^¥^	Mean ± SD			*p*^¥^	Mean ± SD				*p*^¥^
Age (y)	60.1 ± 3.7	62.0 ± 4.6	63.6 ± 5.3	**<0.001**	62.7 ± 5.3	61.9 ± 4.0	61.3 ± 4.1	**<0.01**	61.7 ± 5.0	62.3 ± 4.9	62.2 ± 4.6	60.6 ± 4.0	0.47
Week of blood sampling	111 ± 62	141 ± 74	164 ± 83	**<0.001**	191 ± 64	131 ± 74	101 ± 64	**<0.001**	138 ± 70	155 ± 77	129 ± 69	90 ± 75	**<0.001**
Waist-to-hip ratio^§^	0.78 ± 0.05	0.79 ± 0.05	0.81 ± 0.06	**<0.001**	0.79 ± 0.06	0.79 ± 0.05	0.79 ± 0.06	**0.01**	0.79 ± 0.06	0.79 ± 0.06	0.79 ± 0.05	0.80 ± 0.06	**0.04**
	N (%)				N (%)				N (%)				
**Education**													
Primary school	122 (44.2)	153 (50.7)	167 (54.6)		131 (46.1)	138 (48.8)	173 (54.6)		105 (44.9)	221 (51.2)	60 (54.1)	56 (52.3)	
Elementary school	100 (36.2)	82 (27.2)	96 (31.3)		91 (32.0)	97 (34.3)	90 (28.4)		82 (35.0)	134 (31.0)	32 (28.8)	30 (28.0)	
High school	13 (4.7)	19 (6.3)	15 (4.9)		20 (7.1)	14 (4.9)	13 (4.1)		14 (6.0)	23 (5.3)	4 (3.6)	6 (5.7)	
University	41 (14.9)	48 (15.8)	28 (9.2)	**0.03**	42 (14.8)	34 (12.0)	41 (12.9)	0.29	33 (14.1)	54 (12.5)	15 (13.5)	15 (14.0)	0.86
**Smoking status**													
Never smoker	138 (49.8)	151 (50.2)	169 (55.2)		145 (50.9)	144 (50.9)	169 (53.5)		124 (53.0)	223 (51.6)	50 (45.1)	61 (57.0)	
Former smoker	81 (29.3)	79 (26.2)	80 (26.2)		77 (27.0)	85 (30.0)	78 (24.7)		68 (29.1)	113 (26.2)	34 (30.6)	25 (23.4)	
Active smoker	58 (20.9)	71 (23.6)	57 (18.6)	0.48	63 (22.1)	54 (19.1)	69 (21.8)	0.63	42 (17.9)	96 (22.2)	27 (24.3)	21 (19.6)	0.52
**Alcohol**													
Zero consumers	25 (9.0)	26 (8.6)	26 (8.5)		28 (9.8)	26 (9.2)	23 (7.3)		15 (6.4)	49 (11.4)	8 (7.2)	5 (4.7)	
Low (<15 g/d)	209 (75.5)	238 (78.8)	239 (78.1)		212 (74.4)	222 (78.4)	252 (79.5)		190 (80.8)	328 (75.9)	84 (75.7)	84 (78.5)	
Medium (15–30 g/d)	42 (15.1)	35 (11.6)	37 (12.1)		42 (14.7)	33 (11.7)	39 (12.3)		26 (11.1)	52 (12.0)	19 (17.1)	17 (15.9)	
High (>30 g/d)	1 (0.4)	3 (1.0)	4 (1.3)	0.75	3 (1.1)	2 (0.7)	3 (0.9)	0.79	4 (1.7)	3 (0.7)	0 (0.0)	1 (0.9)	0.13
**Leisure time PA**													
Tertile 1	80 (29.4)	98 (33.2)	101 (33.2)		93 (32.8)	77 (27.9)	109 (34.9)		69 (30.4)	136 (31.7)	33 (30.0)	41 (39.1)	
Tertile 2	96 (35.3)	93 (31.5)	103 (33.9)		95 (33.6)	99 (35.9)	98 (31.4)		71 (31.3)	148 (34.5)	42 (38.2)	31 (29.5)	
Tertile 3	96 (35.3)	104 (35.3)	100 (32.9)	0.77	95 (33.6)	100 (36.2)	105 (33.7)	0.46	87 (38.3)	145 (33.8)	35 (31.8)	33 (31.4)	0.52
**BMI**													
Normal weight (<25)	144 (52.0)	140 (46.5)	122 (39.9)		149 (52.3)	135 (47.7)	122 (38.6)		104 (44.3)	206 (47.8)	49 (44.2)	47 (43.9)	
Overweight (25–30)	99 (35.7)	111 (36.9)	132 (43.1)		103 (36.1)	108 (38.2)	131 (41.5)		97 (41.3)	165 (38.3)	41 (36.9)	39 (36.5)	
Obese (>30)	34 (12.3)	50 (16.6)	52 (17.0)	**0.05**	33 (11.6)	40 (14.1)	63 (19.9)	**<0.01**	34 (14.4)	60 (13.9)	21 (18.9)	21 (19.6)	0.65
**Parity**													
0	29 (10.9)	40 (13.6)	30 (9.9)		43 (15.2)	30 (11.0)	26 (8.4)		24 (10.4)	53 (12.5)	14 (12.8)	8 (7.9)	
1	62 (23.2)	60 (20.4)	63 (20.8)		62 (21.9)	60 (22.0)	63 (20.5)		50 (21.6)	85 (20.1)	28 (25.7)	22 (21.8)	
2	113 (42.3)	115 (39.1)	123 (40.6)		114 (40.3)	103 (37.7)	134 (43.5)		98 (42.4)	174 (41.1)	39 (35.9)	40 (39.6)	
3	37 (13.9)	49 (16.7)	56 (18.5)		43 (15.2)	50 (18.3)	49 (15.9)		31 (13.5)	76 (18.0)	14 (12.8)	21 (20.9)	
≥ 4	26 (9.7)	30 (10.2)	31 (10.2)	0.78	21 (7.4)	30 (11.0)	36 (11.7)	0.19	28 (12.1)	35 (8.3)	14 (12.8)	10 (9.8)	0.52
**MHT**													
No use	195 (79.3)	216 (77.1)	243 (84.1)		201 (72.8)	213 (82.2)	240 (85.7)		172 (77.8)	321 (79.7)	97 (90.7)	64 (76.2)	
Current Use	51 (20.7)	64 (22.9)	46 (15.9)	0.10	75 (27.2)	46 (17.8)	40 (14.3)	**<0.001**	49 (22.2)	82 (20.3)	10 (9.3)	20 (23.8)	**0.03**

^¥^*p*-values were calculated with ANOVA and Chi-square.

ANOVA was used to calculate level differences across levels of biomarkers of inflammation (adjusting for age and week of blood sampling^§^). Chi-square was used to calculate proportion differences.

Since the week of blood sampling differ across most of the biomarker tertiles (Table 2) one could suspect an effect of storage time on biomarker levels; a higher week number (shorter storage time) is seen in the highest tertiles of ox-LDL. But in IL-1β and TNF-α, an opposite pattern is seen. This observation could be partially due to older age effect [35], which in our study also is seen in the same direction across tertiles, and is correlated to the week of blood sampling (r = 0.33, *p*<0.001). However, when adjusting for age, we still see a significant, but less pronounced, difference between week number across different categories of ox-LDL, IL-1β, and TNF-α. By design, the participants that joined the MDC study in the last 2 years of baseline measurements (1995–96) were older (i.e., the mean age of 65 years, compared to mean age of 60–61 years in the first 4 years, *p*<0.001). When repeating all analyses excluding the older age group (>70 years), the results remained unchanged. Nevertheless, we adjusted for week of blood sampling in all analyses. Although WBC, lymphocytes and neutrophils were analyzed at baseline (i.e., with no storage time), these biomarkers also seemed to be affected by week of blood sampling (S1 Table). These associations are probably introduced by the matching procedure (i.e., age and week of blood sampling are matching variables).

### Correlations between inflammation markers

The correlations between adiposity/obesity measures and the inflammation markers are described in Table 3. BMI and WHR were differently associated with the biomarkers; BMI did not correlate with WBC, neutrophils, or with IL-1β, whereas WHR was significantly associated with all biomarkers.

**Table 3:** Correlation of inflammation markers among breast cancer controls (n = 885) in the Malmö Diet and Cancer cohort.

	BMI	WHR	Ox-LDL	IL-1β	IL-6	IL-8	TNF-α	WBC	NEUT
BMI	1								
WHR	0.45***	1							
Ox-LDL	0.12***	0.22***	1						
IL-1β	0.06	0.10**	0.02	1					
IL-6	0.28***	0.15***	0.04	0.15***	1				
IL-8	0.10**	0.09**	0.08*	0.05	0.31***	1			
TNF-α	0.18***	0.08*	0.09**	0.12***	0.51***	0.57***	1		
WBC	0.05	0.16***	0.03	0.07	0.25***	-0.12**	0.01	1	
NEUT	0.02	0.13***	-0.01	0.10**	0.25***	-0.16***	-0.02	0.91***	1
LYMP	0.09**	0.14***	0.07*	-0.04	0.10**	0.01	0.00	0.63***	0.30***

Partial correlations adjusted for age and week of blood sampling. BMI, body mass index, WHR, waist to hip ration, IL, interleukin, TNF, tumor necrosis factor, WBC, white blood cell count, NEUT, neutrophils, LYMP, lymphocytes. All variables were ln-transformed.

*p<0.05,

**p<0.01,

***p<0.001

The strongest correlations among biomarkers were observed between WBC and neutrophils, and with WBC and lymphocytes. Ox-LDL was weakly correlated with IL-8, TNF-α and lymphocytes. On the other hand, IL-6 was positively correlated with all inflammation markers, but not with ox-LDL. TNF-α was correlated with all inflammation markers except the WBC and its subtypes.

### Inflammation and breast cancer

Of all inflammatory markers investigated, we found that ox-LDL, IL-1β, and TNF-α were significantly associated with breast cancer (model I) when adjusting for matching factors (Table 4). Participants in the higher tertiles of ox-LDL and TNF-α were at lower risk of breast cancer compared to the first tertile, whereas the opposite was seen for IL-1β. The observed associations for ox-LDL, IL-1β, and TNF-α remained significant also after adjusting for possible confounders: BMI, WHR, MHT, parity, smoking, alcohol, PA and education (model IV) (Table 4). When all inflammation markers, significantly associated with breast cancer, were included together in the same model (model I) the risk estimates remained similar (i.e., the effects seemed to be independent of each other), but the association was only borderline significant for IL-1β (p = 0.05). The observed risk estimates, that is OR (95% CI) of the highest tertiles, were 0.73 (0.53–0.99), 1.51 (0.99–2.30), and 0.60 (0.41–0.86) for ox-LDL, IL-1β and TNF-α, respectively.

**Table 4:** Association between inflammation markers and the risk of post-menopausal breast cancer in the Malmö Diet and Cancer cohort.

			Model I	Model II	Model III	Model IV
	Median	Case/control	OR (95% CI)			
**Ox-LDL** (U/l)						
Tertile 1 (n = 445)	46.6	168/277	1 (reference)	1 (reference)	1 (reference)	1 (reference)
Tertile 2 (n = 442)	62.0	140/302	**0.75** (0.57–1.00)	**0.74** (0.56–0.99)	**0.65** (0.47–0.90)	**0.65** (0.47–0.90)
Tertile 3 (n = 444)	85.9	138/306	**0.71** (0.52–0.96)	**0.69** (0.51–0.94)	**0.65** (0.46–0.91)	**0.63** (0.45–0.89)
*p trend*			**0.03**	**0.02**	**0.01**	**0.01**
**IL-1β** (pg/ml)						
0 (n = 340)	0	105/235	1 (reference)	1 (reference)	1 (reference)	1 (reference)
<LLOD (n = 640)	0.10	208/432	1.08 (0.80–1.44)	1.08 (0.80–1.45)	1.08 (0.79–1.45)	1.09 (0.78–1.51)
Cat 1 (n = 176)	0.22	65/111	1.45 (0.94–2.24)	1.45 (0.93–2.25)	1.58 (0.99–2.53)	**1.62** (1.00–2.62)
Cat 2 (n = 175)	0.76	68/107	**1.52** (1.01–2.30)	1.51 (0.99–2.28)	**1.64** (1.02–2.65)	**1.71** (1.05–2.79)
*p trend*			**0.02**	**0.03**	**0.02**	**0.01**
**IL-6** (pg/ml)						
Tertile 1 (n = 446)	0.80	159/287	1 (reference)	1 (reference)	1 (reference)	1 (reference)
Tertile 2 (n = 442)	1.45	138/304	0.80 (0.59–1.07)	0.76 (0.56–1.03)	0.76 (0.55–1.05)	0.75 (0.54–1.05)
Tertile 3 (n = 443)	2.62	149/294	0.87 (0.64–1.18)	0.80 (0.58–1.10)	0.83 (0.58–1.18)	0.80 (0.56–1.15)
*p trend*			0.40	0.19	0.32	0.25
**IL-8** (pg/ml)						
Tertile 1 (n = 446)	3.10	149/297	1 (reference)	1 (reference)	1 (reference)	1 (reference)
Tertile 2 (n = 445)	5.12	153/292	1.06 (0.75–1.50)	1.04 (0.73–1.48)	1.08 (0.74–1.57)	1.13 (0.77–1.65)
Tertile 3 (n = 440)	7.61	144/296	0.97 (0.67–1.41)	0.96 (0.66–1.41)	1.05 (0.69–1.59)	1.09 (0.71–1.66)
*p trend*			0.80	0.78	0.86	0.76
**TNF-α** (pg/ml)						
Tertile 1 (n = 446)	1.40	161/285	1 (reference)	1 (reference)	1 (reference)	1 (reference)
Tertile 2 (n = 439)	2.25	156/283	0.86 (0.62–1.19)	0.82 (0.59–1.14)	0.93 (0.65–1.33)	0.91 (0.63–1.32)
Tertile 3 (n = 446)	3.28	129/317	**0.60** (0.42–0.86)	**0.56** (0.38–0.81)	**0.65** (0.42–0.98)	**0.65** (0.43–0.99)
*p trend*			**0.01**	**0.01**	**0.03**	**0.04**
**WBC** (count)						
Tertile 1 (n = 448)	4.90	145/303	1 (reference)	1 (reference)	1 (reference)	1 (reference)
Tertile 2 (n = 447)	6.00	152/295	1.07 (0.81–1.42)	1.07 (0.80–1.42)	1.03 (0.75–1.41)	1.01 (0.73–1.40)
Tertile 3 (n = 436)	7.60	149/287	1.08 (0.81–1.44)	1.06 (0.80–1.41)	0.96 (0.70–1.32)	0.93 (0.67–1.30)
*p trend*			0.59	0.69	0.81	0.66
**Lymphocytes** (count)						
Tertile 1 (n = 444)	1.50	142/302	1 (reference)	1 (reference)	1 (reference)	1 (reference)
Tertile 2 (n = 445)	1.90	148/311	0.83 (0.62–1.10)	0.81 (0.61–1.08)	0.77 (0.56–1.05)	0.79 (0.57–1.09)
Tertile 3 (n = 427)	2.50	156/271	0.97 (0.74–1.27)	0.94 (0.72–1.24)	0.97 (0.71–1.30)	0.94 (0.68–1.28)
*p trend*			0.81	0.66	0.76	0.62
**Neutrophils** (count)						
Tertile 1 (n = 486)	2.70	171/315	1 (reference)	1 (reference)	1 (reference)	1 (reference)
Tertile 2 (n = 392)	3.60	121/271	1.00 (0.75–1.33)	1.00 (0.75–1.33)	1.01 (0.74–1.38)	1.02 (0.74–1.40)
Tertile 3 (n = 452)	4.80	154/298	1.21 (0.91–1.62)	1.20 (0.90–1.60)	1.06 (0.77–1.46)	1.04 (0.74–1.46)
*p trend*			0.19	0.23	0.72	0.82

Breast Cancer risk (OR) were calculated using conditional logistic regression, with the lowest category (Tertile 1) as the reference group. Adjustment models included: age and week of blood sampling (model I), BMI (model II), WHR, MHT and parity (model III), smoking, alcohol, PA and education (model IV).

Associations remained similar for ox-LDL and TNF-α when excluding those diagnosed within the 1^st^ of follow-up (n = 45), and were slightly attenuated with the exclusion of 1^st^ and 2^nd^ (n = 64) year of follow-up. When excluding women diagnosed until the 3^rd^ year of follow-up (n = 83), most associations with breast cancer were attenuated (i.e., non-significant), but the magnitude of the estimates remained. Associations with IL-1β were no longer significant when excluding cases diagnosed during the first year of follow-up.

When adding season to the models in sensitivity analysis, no changes in the estimates were seen. Season was also related to the year of the baseline examination; participants in the later 2 years (1995–96) were more likely to be measured during the fall or winter. Finally, when accounting for batch effect, results (point estimates and p-values) remained virtually the same.

In *ad hoc* analysis, when labeling the missing values for MHT and parity as such and studying the association between inflammation markers and breast cancer risk for 1300 women, we observed that the associations in the highest category of IL-1β in models III and IV were no longer significant; 1.43 (0.94–2.19), 1.53 (0.98–2.37) respectively. However, the p-value for trend remained significant (0.04 and 0.02) as well as for category 1 of IL-1β, 1.61 (1.02–2.57). In addition, an inverse and significant association with breast cancer was observed for the second tertile of IL-6, 0.72 (0.53–0.98).

### Obesity and biomarkers

Interactions between obesity measures and biomarkers were examined with tertile variables entered as continuous variables in the fully adjusted model using unconditional logistic regression. Interactions were not significant; ox-LDL x WHR (*p* = 0.57), ox-LDL x BMI (*p* = 0.63), IL-1β x WHR (*p* = 0.09), IL-1β x BMI (*p* = 0.79), TNF-α x WHR (*p* = 0.11), and TNF-α x BMI (*p* = 0.49).

## Discussion

In this nested case-control study, we analyzed several biomarkers of inflammation that is ox-LDL, IL-1β, IL-6, TNF-α, WBC, neutrophils, and lymphocytes, and found significant associations between ox-LDL, IL-1β, and TNF-α and post-menopausal breast cancer. The ox-LDL and TNF-α variables showed inverse associations, while IL-1β was associated with increased breast cancer risk. These associations remained after adjusting for risk factors and potential confounders.

A recent review and meta-analysis supports the role of chronic inflammation in breast cancer development, but also highlighted the importance of studying potential confounders and modifying factors in more detail [36]. The meta-analysis was based on twelve studies and reported a 7% increase in breast cancer risk when the concentration of circulating C-reactive protein (CRP) was doubled.

To our knowledge, the association between several biomarkers of inflammation, other than CRP and breast cancer risk has not been extensively examined previously. This case-control study nested within a cohort allowed us to analyze several inflammation markers for the same individuals, as opposed to having more individuals and just one biomarker. The study has a prospective design (i.e., dietary data and blood samples collected long before the disease was diagnosed), is well powered and population based [27]. We also had access to a wide range of information about lifestyle factors, which allowed us to adjust for many of the known risk factors for post-menopausal breast cancer.

We analyzed several biomarkers, in order to obtain a broader view of inflammation in our study sample, and Table 3 shows us, that certain patterns emerge. Of note is that IL-6 according to previous reports may act both as an anti-inflammatory and a pro-inflammatory cytokine and has previously been implicated with breast cancer [37, 38]. In the present study, IL-6 was associated with all the other biomarkers, except with ox-LDL. This prompts us to speculate that each of these biomarkers is involved in different processes and possibly at different stages of the disease development, and one of them will not necessarily alone identify the degree of inflammation. In fact, WBC, which is recognized as a strong indicator of inflammation promoting cardiovascular events [39], was not correlated with ox-LDL, IL-1β and TNF-α, whereas these biomarkers yielded significant associations with breast cancer in the present study.

Oxidized-LDL is a marker of oxidative stress [40–42], and its role in atherosclerosis is well established in the literature [43–45]. Our results (Table 2) support previous reports showing that ox-LDL levels naturally increase with age [35, 46]. Although epidemiological studies have shown high levels of ox-LDL to be associated with increased risk of certain types of cancer [47, 48], a dual tumor protective role of ox-LDL has been reported in studies of cancer cells (in vitro); a cytotoxic effect that activated apoptosis (leading to cellular death) and autophagy (a pro-survival mechanism) [49]. It has been suggested that normal breast cells readily internalize ox-LDL, leading to the increase of proliferative and pro-inflammatory signaling, setting the stage for breast cancer development [47]. Because the uptake of cholesterol is greater in breast cancer cells than in normal cells, lower circulating levels of total cholesterol (or its fractions) could be associated with increased breast cancer risk, probably indicating that active undiagnosed tumors are in place (i.e., reverse causality). Two recent meta-analyses of blood lipids and breast cancer risk support this hypothesis [50, 51]; *Ni H*., *et al*., reported a significant reduction of total breast cancer risk (both pre- and post-menopausal) for higher levels of triglycerides and in subsequent sub-group analysis an inverse association was observed for higher levels of HDL (only among post-menopausal women). *Touvier*, *M*. and colleagues observed an inverse association between total cholesterol and the risk of breast cancer (both in pre- and post-menopausal status). However, in the current study we cannot exclude that other underlying mechanisms are the culprits for both the increased breast cancer risk, and lower levels of ox-LDL.

TNF-α is produced by several cells such as macrophages, neutrophils, and lymphocytes. It is considered an acute phase reaction cytokine involved in systemic inflammation [52], and acts as a cytotoxic factor in cancer cells [53]. It is also closely related to obesity (especially abdominal fat in the metabolic syndrome [54]), and was in our study associated with WHR, r = 0.39 (*p*<0.001). TNF-α is a highly pleiotropic cytokine, and dual roles have been reported in carcinogenesis [21, 25, 55]. High concentration of this cytokine is associated with the ability to kill tumor cells, but also with the stimulation of fibroblasts or tumor cell growth [56]. Additionally, high concentrations of TNF-α seem to be associated with increased activity of aromatase [57]. The few existing epidemiological studies have reported no significant associations between levels of circulating TNF-α and breast cancer risk [58, 59].

The IL-1β was the only cytokine in our study where higher levels were associated with increased risk of post-menopausal breast cancer. It has a role in cell proliferation and differentiation, but an involvement of IL-1β in apoptosis has also been described [60]. The presence of IL-1β and IL-1α in tumor cells in patients with invasive breast cancer has also been described [61]. However, it was previously hypothesized that a coordinated expression of IL-1β and TNF-α would be important for the progression of the disease [62]. In our study, we see opposite effects in these two biomarkers which are positively correlated with each other. It is possible that the coordinated expression of IL-1β and TNF-α is more important in the progression phase than in oncogenesis. Also, the non-significant association observed after excluding cases diagnosed during the 1^st^ year of follow-up might be indicative of reverse causality. However, loss of power as an alternative possibility for the attenuated association cannot be excluded, since there is a significant reduction in size in the higher categories, due to the fact that 74% of the IL-1β levels were below LLOD; this is also a major limitation.

One single measurement of these biomarkers was previously found to be highly reliable within a period of few months, in sub-samples drawn from the MDC cohort [63]. However, some of these biomarkers have limitations during longer time periods [64]. It is possible that biomarker levels at baseline are not representative of the long-term exposure. Cancer development is believed to evolve during many years. Since inflammation potentially could play an important part during certain key periods [9], it might be impossible to discern the specific biomarkers as the process is time point specific for each individual. In fact, evidence in many of these biomarkers is currently controversial regarding cancer development [25], and conflicting findings might result from undetected underlying tumors.

In order to obtain a clearer picture, future studies with continuous evaluation of the inflammation levels over a long period are required. It is probably easier to establish a relationship between inflammation and CVD, as disease progression is faster and inflammation biomarkers, such as C-reactive protein, are well established [65]. The lack of information on biomarker levels during a longer time period is the major limitation of this study.

Another factor that could have influenced our results is that systemic inflammation might not be as important as local inflammation, in the development of breast cancer. In fact, the release of inflammatory mediators, that will increase aromatase expression (and consequently the production of estrogens), takes place in the adipocytes close to the ducts of the breast tissue [66]. This might not be reflected in circulatory levels.

We did not observe any interaction between obesity/adiposity and the inflammation biomarkers in relation to breast cancer. Previous reports from the MDC cohort point to the importance of these obesity/adiposity measures in breast cancer risk [67, 68], but according to our observations the associations between inflammation biomarkers and breast cancer seem to be largely independent from obesity. It is possible, however, that due to the small sample size in the present study we did not have enough power to detect associations in the interaction analysis.

It is accepted that cancer development and progression increase inflammation [69]. Hence the sensitivity analyses gradually excluded women diagnosed within the 1^st^ to the 3^rd^ years of follow up. When excluding women diagnosed within the first 3 years of follow up all associations were attenuated, but the magnitude of the estimates remained. This observation points to the loss of power, and, probably excludes the possibility for reverse causality for ox-LDL and TNF-α.

In breast tumors, which are estrogen responsive (ER+), inflammation could potentially contribute to the development of tumors through the induction of aromatase action [7]. It is, however, possible that the role of inflammation in post-menopausal breast cancer was, in this study, overshadowed by the hormonal factors. In addition, it is possible that inflammation may play a role in the tumor types non-responsive to hormones; these tumors are more common in pre-menopausal breast cancer cases [13, 70]. Considering that breast cancer is a heterogeneous disease, the inability in this study to examine the breast cancer risk separately for different tumor types is an important limitation.

In conclusion, our results do not support the hypothesis that low-grade systemic inflammation is associated with increased risk of post-menopausal breast cancer. According to the results demonstrated in this study, the association between breast cancer and inflammatory markers, and breast cancer and obesity indicators appear independent of each other. These observations prompt confirmation in larger studies with routine analyses of inflammation markers during longer periods, and also with information on different tumor types.

## Supporting Information

S1 Table Characteristics of breast cancer controls across levels of several inflammation markers (WBC, lymphocytes and neutrophils) in the Malmö Diet and Cancer cohort. (DOCX)

S2 Table Characteristics of breast cancer controls across levels of several inflammation markers (IL-6 and IL-8) in the Malmö Diet and Cancer cohort. (DOCX)

## Abbreviations

BMI: body mass index
CI: confidence intervals
CVD: cardiovascular disease
IL: interleukin
MDC: Malmö Diet and Cancer
OR: odds ratio
ox-LDL: oxidized low-density lipoprotein
TNF-α: tumor necrosis factor alpha
WBC: white blood cell
WHR: waist-to-hip ratio
