# Supplementary material for: Low-Grade Inflammation, Oxidative Stress and Risk of Invasive Post-Menopausal Breast Cancer - A Nested Case-Control Study from the Malmö Diet and Cancer Cohort
Source: PLoS One. 2016 Jul 8;11(7):e0158959. doi: 10.1371/journal.pone.0158959 (PMC4938491; doi:10.1371/journal.pone.0158959)
Supplement: S1 Table — (DOCX) [file pone.0158959.s001.docx]

**S1 Table**  – Characteristics of breast cancer controls across levels of several inflammation markers (WBC, lymphocytes and neutrophils) in the Malmö Diet and Cancer cohort

|  |  | **WBC** |  |  | **Lymphocytes** | | |  | **Neutrophils** | |  |  |
| --- | --- | --- | --- | --- | --- | --- | --- | --- | --- | --- | --- | --- |
|  | Tertile 1  (n=312) | Tertile 2  (n=304) | Tertile 3  (n=294) |  | Tertile 1  (n=324) | Tertile 2  (n=278) | Tertile 3  (n=307) |  | Tertile 1  (n=310) | Tertile 2  (n=320) | Tertile 3  (n=279) |  |
|  | Mean ± SD | | | *p** | Mean ± SD | | | *p** | Mean ± SD | | | *p** |
| Age (y) | 61.1 ± 4.0 | 62.2 ± 5.0 | 62.7 ± 5.1 | **<0.01** | 61.7 ± 4.4 | 62.0 ± 4.8 | 62.2 ± 5.1 | 0.35 | 61.1 ± 4.2 | 62.1 ± 4.9 | 62.9 ± 5.1 | **<0.01** |
| Week of blood sampling | 123 ± 58 | 139 ± 80 | 155 ± 79 | **<0.01** | 128 ± 71 | 143 ± 77 | 146 ± 82 | **<0.05** | 127 ± 74 | 138 ± 76 | 152 ± 79 | **<0.01** |
| Waist-to-hip ratio^§^ | 0.75 ± 0.05 | 0.79 ± 0.05 | 0.81 ± 0.06 | **<0.01** | 0.78 ± 0.05 | 0.79 ± 0.06 | 0.80 ± 0.06 | **<0.01** | 0.78 ± 0.05 | 0.79 ± 0.05 | 0.80 ± 0.06 | **<0.01** |
|  | N (%) | | |  | N (%) | | |  | N (%) | | |  |
| **Education** |  |  |  |  |  |  |  |  |  |  |  |  |
| Primary school | 154 (49.4) | 142 (46.7) | 158 (53.9) |  | 161 (49.7) | 131 (47.4) | 162 (52.9) |  | 148 (47.7) | 162 (50.6) | 144 (51.8) |  |
| Elementary school | 95 (30.4) | 101 (33.2) | 91 (31.1) |  | 95 (29.3) | 96 (34.5) | 95 (31.0) |  | 94 (30.3) | 99 (30.9) | 93 (33.5) |  |
| High school | 17 (5.4) | 19 (6.3) | 11 (3.8) |  | 21 (6.5) | 15 (5.4) | 11 (3.6) |  | 14 (4.5) | 21 (6.6) | 12 (4.3) |  |
| University | 46 (14.7) | 42 (13.8) | 33 (11.3) | 0.50 | 47 (14.5) | 36 (12.9) | 38 (12.4) | 0.49 | 54 (17.4) | 38 (11.9) | 29 (10.4) | 0.17 |
| **Smoking status** |  |  |  |  |  |  |  |  |  |  |  |  |
| Never smoker | 185 (59.3) | 161 (53.0) | 127 (43.3) |  | 195 (60.2) | 138 (49.6) | 139 (45.4) |  | 180 (58.3) | 172 (53.8) | 120 (43.0) |  |
| Former smoker | 93 (29.8) | 88 (28.9) | 64 (21.8) |  | 86 (26.5) | 86 (30.9) | 73 (23.9) |  | 93 (30.1) | 87 (27.2) | 65 (23.3) |  |
| Active smoker | 34 (10.9) | 55 (18.1) | 102 (34.8) | **<0.01** | 43 (13.3) | 54 (19.4) | 94 (30.7) | **<0.01** | 36 (11.7) | 61 (19.1) | 94 (33.7) | **<0.01** |
| **Alcohol consumption** |  |  |  |  |  |  |  |  |  |  |  |  |
| Zero consumers | 20 (6.4) | 27 (8.9) | 31 (10.5) |  | 26 (8.0) | 19 (6.8) | 32 (10.4) |  | 17 (5.5) | 28 (8.8) | 32 (11.5) |  |
| Low (<15 g/d) | 248 (79.5) | 229 (75.3) | 230 (78.2) |  | 257 (79.3) | 211 (75.9) | 239 (77.9) |  | 251 (81.0) | 242 (75.6) | 214 (76.7) |  |
| Medium (15-30 g/d) | 41 (13.1) | 44 (14.5) | 32 (10.9) |  | 36 (11.1) | 47 (16.9) | 34 (11.1) |  | 41 (13.2) | 44 (13.8) | 32 (11.5) |  |
| High (>30 g/d) | 3 (1.0) | 4 (1.3) | 1 (0.3) | 0.36 | 5 (1.5) | 1 (0.4) | 2 (0.7) | 0.11 | 1 (0.3) | 6 (1.9) | 1 (0.4) | **<0.05** |
| **Leisure time PA** |  |  |  |  |  |  |  |  |  |  |  |  |
| Tertile 1 | 86 (28.0) | 87 (29.0) | 114 (39.4) |  | 91 (28.6) | 80 (29.0) | 116 (38.5) |  | 87 (28.6) | 94 (29.7) | 103 (38.7) |  |
| Tertile 2 | 107 (34.9) | 103 (34.3) | 90 (31.1) |  | 102 (32.1) | 100 (36.2) | 97 (32.2) |  | 105 (34.5) | 116 (36.6) | 78 (28.5) |  |
| Tertile 3 | 114 (37.1) | 110 (36.7) | 85 (29.4) | **<0.05** | 125 (39.3) | 96 (34.8) | 88 (29.2) | **<0.05** | 112 (36.8) | 107 (33.8) | 90 (32.8) | 0.05 |
| **BMI** |  |  |  |  |  |  |  |  |  |  |  |  |
| Normal weight (>25) | 148 (47.4) | 147 (48.5) | 125 (42.5) |  | 169 (52.2) | 123 (44.2) | 127 (41.5) |  | 145 (46.8) | 149 (46.7) | 125 (44.8) |  |
| Overweight (25-30) | 123 (39.4) | 115 (38.0) | 111 (37.8) |  | 112 (34.6) | 110 (39.6) | 127 (41.5) |  | 124 (40.0) | 123 (38.6) | 102 (36.6) |  |
| Obese (<30) | 41 (13.1) | 41 (13.5) | 58 (19.7) | 0.15 | 43 (13.3) | 45 (16.2) | 52 (17.0) | 0.10 | 41 (13.2) | 47 (14.7) | 52 (18.6) | 0.47 |
| **Parity** |  |  |  |  |  |  |  |  |  |  |  |  |
| 0 | 29 (9.4) | 30 (10.1) | 42 (14.7) |  | 30 (9.4) | 33 (12.1) | 38 (12.8) |  | 29 (9.5) | 34 (10.9) | 38 (13.9) |  |
| 1 | 68 (22.1) | 51 (17.2) | 75 (26.3) |  | 77 (24.1) | 59 (21.7) | 58 (19.6) |  | 56 (18.4) | 70 (22.5) | 68 (24.9) |  |
| 2 | 128 (41.6) | 138 (46.6) | 195 (33.3) |  | 134 (41.9) | 116 (42.6) | 110 (37.2) |  | 130 (42.8) | 132 (42.4) | 98 (35.9) |  |
| 3 | 51 (16.6) | 43 (29.7) | 51 (17.9) |  | 48 (15.0) | 37 (13.6) | 60 (20.3) |  | 55 (18.1) | 44 (14.1) | 46 (16.8) |  |
| ≥ 4 | 32 (10.4) | 34 (11.5) | 32 (7.7) | **<0.05** | 31 (9.7) | 27 (9.9) | 30 (10.1) | 0.35 | 34 (11.2) | 31 (10.0) | 23 (8.4) | 0.26 |
| **MHT** |  |  |  |  |  |  |  |  |  |  |  |  |
| No use | 235 (82.5) | 215 (77.3) | 222 (81.0) |  | 238 (80.1) | 201 (77.9) | 233 (82.6) |  | 232 (82.6) | 231 (78.3) | 209 (80.1) |  |
| Current Use | 50 (17.5) | 63 (22.7) | 52 (19.0) | 0.29 | 59 (19.9) | 57 (22.1) | 49 (17.7) | 0.39 | 49 (17.4) | 67 (21.7) | 52 (19.9) | 0.44 |

^¥^*p*-values were calculated with ANOVA and Chi-square. ANOVA was used to calculate level differences across levels of biomarkers of inflammation (adjusting for age and week of blood sampling^§^). Chi-square was used to calculate proportion differences.
