# Supplementary material for: Low-Grade Inflammation, Oxidative Stress and Risk of Invasive Post-Menopausal Breast Cancer - A Nested Case-Control Study from the Malmö Diet and Cancer Cohort
Source: PLoS One. 2016 Jul 8;11(7):e0158959. doi: 10.1371/journal.pone.0158959 (PMC4938491; doi:10.1371/journal.pone.0158959)
Supplement: S2 Table — (DOCX) [file pone.0158959.s002.docx]

**S2 Table** – Characteristics of breast cancer controls across levels of several inflammation markers (IL-6 and IL-8) in the Malmö Diet and Cancer cohort

|  |  | **IL-6** |  |  |  | **IL-8** |  |  |
| --- | --- | --- | --- | --- | --- | --- | --- | --- |
|  | Tertile 1  (n=293) | Tertile 2  (n=314) | Tertile 3  (n=303) |  | Tertile 1  (n=303) | Tertile 2  (n=299) | Tertile 3  (n=308) |  |
|  | Mean ± SD | | | *p** | Mean ± SD | | | *p** |
| Age (y) | 61.9 ± 5.2 | 62.0 ± 4.7 | 63.6 ± 5.2 | 0.98 | 63.5 ± 5.4 | 61.2 ± 4.5 | 61.3 ± 3.9 | **<0.01** |
| Week of blood sampling | 168 ± 78 | 129 ± 74 | 121 ± 70 | **<0.01** | 200 ± 58 | 116 ± 70 | 99 ± 58 | **<0.01** |
| Waist-to-hip ratio^§^ | 0.78 ± 0.05 | 0.79 ± 0.05 | 0.80 ± 0.06 | **<0.01** | 0.79 ± 0.06 | 0.79 ± 0.05 | 0.80 ± 0.06 | **<0.05** |
|  | N (%) | | |  | N (%) | | |  |
| **Education** |  |  |  |  |  |  |  |  |
| Primary school | 128 (43.7) | 150 (47.9) | 176 (58.1) |  | 139 (46.0) | 149 (49.8) | 166 (53.9) |  |
| Elementary school | 109 (37.2) | 100 (31.9) | 78 (25.7) |  | 110 (36.4) | 93 (31.1) | 84 (27.3) |  |
| High school | 17 (5.8) | 15 (4.8) | 15 (5.0) |  | 15 (5.0) | 18 (6.0) | 14 (4.5) |  |
| University | 39 (13.3) | 48 (15.3) | 34 (11.2) | **<0.05** | 38 (12.6) | 39(13.0) | 44 (14.5) | 0.32 |
| **Smoking status** |  |  |  |  |  |  |  |  |
| Never smoker | 148 (50.5) | 169 (53.8) | 156 (51.7) |  | 170 (56.1) | 149 (49.8) | 154 (50.6) |  |
| Former smoker | 90 (30.7) | 88 (28.0) | 67 (22.2) |  | 77 (25.4) | 91 (30.4) | 77 (25.4) |  |
| Active smoker | 55 (18.8) | 57 (18.2) | 79 (26.2) | **<0.05** | 56 (18.5) | 59 (19.7) | 76 (24.8) | 0.16 |
| **Alcohol consumption** |  |  |  |  |  |  |  |  |
| Zero consumers | 20 (6.8) | 29 (9.2) | 29 (9.6) |  | 29 (9.6) | 21 (7.0) | 28 (9.1) |  |
| Low (<15 g/d) | 216 (73.7) | 245 (78.0) | 246 (81.2) |  | 232 (76.6) | 236 (78.9) | 239 (77.6) |  |
| Medium (15-30 g/d) | 55 (18.8) | 37 (11.8) | 25 (8.3) |  | 39 (12.9) | 39 (13.0) | 39 (12.7) |  |
| High (>30 g/d) | 2 (0.7) | 3 (1.0) | 3 (1.0) | **<0.05** | 3 (1.0) | 3 (1.1) | 2 (0.6) | 0.95 |
| **Leisure time PA** |  |  |  |  |  |  |  |  |
| Tertile 1 | 84 (28.8) | 91 (29.6) | 112 (37.7) |  | 93 (30.9) | 93 (31.5) | 101 (33.7) |  |
| Tertile 2 | 99 (33.9) | 106 (34.5) | 95 (32.0) |  | 95 (31.6) | 109 (36.9) | 96 (32.0) |  |
| Tertile 3 | 109 (37.3) | 110 (35.8) | 90 (30.3) | 0.13 | 113 (37.5) | 93 (31.5) | 103 (34.3) | 0.47 |
| **BMI** |  |  |  |  |  |  |  |  |
| Normal weight (>25) | 173 (59.0) | 141 (44.9) | 106 (35.1) |  | 142 (46.9) | 139 (46.5) | 139 (45.3) |  |
| Overweight (25-30) | 104 (35.5) | 124 (39.5) | 121 (40.1) |  | 121 (39.9) | 124 (41.5) | 104 (33.9) |  |
| Obese (<30) | 16 (5.5) | 49 (15.6) | 75 (24.8) | **<0.001** | 40 (13.2) | 36 (12.0) | 64 (20.8) | **<0.05** |
| **Parity** |  |  |  |  |  |  |  |  |
| 0 | 36 (12.5) | 30 (9.7) | 35 (11.9) |  | 42 (14.0) | 32 (11.0) | 27 (9.1) |  |
| 1 | 64 (22.2) | 63 (20.5) | 67 (22.9) |  | 67 (22.3) | 55 (18.9) | 72 (24.2) |  |
| 2 | 119 (41.3) | 139 (45.1) | 103 (35.2) |  | 125 (41.7) | 124 (42.6) | 112 (37.6) |  |
| 3 | 44 (15.3) | 46 (14.9) | 55 (18.8) |  | 47 (15.7) | 50 (17.2) | 48 (16.1) |  |
| ≥ 4 | 25 (8.7) | 30 (9.7) | 33 (11.3) | 0.44 | 19 (6.3) | 30 (10.3) | 39 (13.1) | 0.10 |
| **MHT** |  |  |  |  |  |  |  |  |
| No use | 207 (75.8) | 219 (75.5) | 246 (89.8) |  | 219 (74.5) | 212 (79.4) | 241 (87.3) |  |
| Current Use | 66 (24.2) | 71 (24.5) | 28 (10.2) | **<0.001** | 75 (25.5) | 55 (20.6) | 35 (12.7) | **<0.01** |

^¥^*p*-values were calculated with ANOVA and Chi-square. ANOVA was used to calculate level differences across levels of biomarkers of inflammation (adjusting for age and week of blood sampling^§^). Chi-square was used to calculate proportion differences.
